# Necrosis-Suppressing Effector Protein ChEC88 Adopts a Novel Structural Motif Conserved Among Genus-Spanning Hemibiotrophic Phytopathogens

**DOI:** 10.3390/plants14162562

**Published:** 2025-08-18

**Authors:** Shinya Ohki, Hiroyuki Takahara, Tomohiro Imamura, Kosei Sakane, Asihan Bai, Kazunori Sasaki, Takumi Nishiuchi, Masashi Mori

**Affiliations:** 1 Center for Nano Materials and Technology (CNMT), Japan Advanced Institute of Science and Technology (JAIST), Nomi 923-1292, Ishikawa, Japan; s2510144@jaist.ac.jp; 2 Department of Bioproduction Science, Ishikawa Prefectural University, Nonoichi 921-8836, Ishikawa, Japan; takahara@ishikawa-pu.ac.jp (H.T.); timamura@ishikawa-pu.ac.jp (T.I.); k-sakane@ishikawa-pu.ac.jp (K.S.); 3 Research Center for Thermotolerant Microbial Resources (RCTMR), Yamaguchi University, Yamaguchi 753-8515, Yamaguchi, Japan; sasaki@yamaguchi-u.ac.jp; 4 Division of Integrated Omics Research, Bioscience Core Facility, Research Center for Experimental Modeling of Human Disease, Kanazawa University, Kanazawa 920-8640, Ishikawa, Japan; tnish9@staff.kanazawa-u.ac.jp; 5 Research Institute for Bioresources and Biotechnology, Ishikawa Prefectural University, Nonoichi 921-8836, Ishikawa, Japan

**Keywords:** ChEC88, effector, *Colletotrichum*, phytopathogenic fungi, hemi-biotrophs, structure

## Abstract

Phytopathogenic fungi secrete numerous effector proteins to disrupt plant defenses. At present, their sequence–structure–function relationships remain poorly understood owing to their diversity. Comprehensive understanding of conserved effectors is necessary to elucidate the molecular relationship between fungi and plants. To fill this research gap, we investigated the *Colletotrichum higginsianum* effector candidate (ChEC)-88 specifically expressed during infection. Notably, similar to the biotrophy-associated secreted protein 3 (BAS3) from *Pyricularia oryzae*, ChEC88 inhibited plant cell death caused by necrosis- and ethylene-inducing peptide 1-like protein (NLP1). Nuclear magnetic resonance analysis results revealed that ChEC88 adopted a novel pseudo two-fold symmetrical three-dimensional structure. Homology modeling suggested that BAS3 exhibited a ChEC88-like conformation despite sharing less than 50% sequence identity. Through PSI-BLAST searches, we found that ChEC88 homologs were conserved in various hemibiotrophic phytopathogenic fungi, including *Colletotrichum*, *P. oryzae*, and *Fusarium* species. Functional assays demonstrated that all of the representative homologs suppressed NLP1-induced plant cell death. Mutation experiments identified the residues critical for ChEC88 function. Overall, our findings suggest that hemibiotrophic phytopathogenic fungi share a conserved immune-suppression strategy mediated by ChEC88-like proteins and that such effectors possibly originated from a common ancestral lineage of phytopathogenic fungi.

## 1. Introduction

Phytopathogenic fungi pose a major threat to agriculture, significantly decreasing crop yields and impacting global food security [1]. Plants have developed the following two-tiered immunity mechanisms to combat such pathogens: pattern-triggered immunity (PTI) and effector-triggered immunity (ETI). PTI is initiated when membrane-associated receptors detect pathogen-associated molecular patterns, triggering plant defense responses [2]. Phytopathogens use effector proteins to suppress PTI, resulting in effector-triggered susceptibility (ETS) [3]. Plants counteract ETS with ETI, mediated by resistance proteins, which detect specific effectors and trigger robust immune responses, such as reactive oxygen species bursts, hypersensitive responses, and systemic acquired resistance. These interactions represent an evolutionary tug-of-war between fungal pathogenicity and plant defense mechanisms [4,5].

Hemibiotrophic pathogens alternate between biotrophic and necrotrophic phases and use distinct effectors to manipulate host immunity and cause infections [6,7]. Pathogens maintain host cell viability to facilitate colonization during the biotrophic phase and destroy the host tissues to extract nutrients during the necrotrophic phase [8]. *Colletotrichum higginsianum*, a model hemibiotrophic fungal pathogen that infects cruciferous plants, is of particular utility in exploring conserved effectors because of its annotated effector gene set and defined expression profiles across infection stages [9]. *Pyricularia oryzae*, which causes rice blasts, and *Fusarium oxysporum*, which causes vascular wilt, secrete diverse effectors to subvert host plant immunity [6,10]. In light of the threat they pose, comparative studies among *C. higginsianum*, *P. oryzae*, and *F. oxysporum* are vital in order to find core and pathogen-specific effector strategies and understand the co-evolution of plants and hemibiotrophic pathogens.

Effector proteins play a central role in fungal pathogen virulence. However, their vast diversity makes the exploration of their infection mechanisms at the molecular level challenging [3,11]. Among the various fungal effectors, conserved effectors have attracted particular interest due to their functional similarities across phylogenetically distant phytopathogens. Evaluation of these factors provides valuable insights into universal virulence strategies and reveals promising targets to induce broad-spectrum resistance in the host. Necrosis-inducing secreted protein 1 (NIS1), a conserved effector in both *Ascomycota* and *Basidiomycota*, is one such well-characterized effector [12,13]. NIS1 is specifically expressed during the biotrophic phase and suppresses host cell death. It disrupts plant immune signaling by targeting immune kinases, such as brassinosteroid insensitive 1-associated receptor kinase 1 (BAK1) and botrytis-induced kinase 1 (BIK1), underscoring the evolutionary importance of conserved effectors in promoting pathogen survival and shaping host adaptation [12].

The necrosis- and ethylene-inducing peptide 1 (Nep1)-like protein (NLP) family, secreted by fungi, bacteria, and oomycetes, is another well-known conserved effector family. NLPs are predominantly expressed during the necrotrophic phase and induce host cell death and ethylene production, facilitating tissue colonization [14,15,16]. Their conservation across microbial lineages highlights their importance as universal virulence factors. Expression of NLP1, a representative NLP family member, peaks during the late infection stage, aligning with the necrotrophic nutrient acquisition needs of the pathogen [9].

In this study, we investigated C. higginsianum effector candidate (ChEC)-88. Notably, ChEC88 shares moderate sequence identity (49%) with the biotrophy-associated secreted protein 3 (BAS3) of *P. oryzae* [9,17,18]; however, to date, its structural and functional properties remain ambiguous. Herein, we report that ChEC88, similar to BAS3, suppressed NLP1-induced plant cell death. Nuclear magnetic resonance (NMR) analysis results revealed that ChEC88 adopted a novel pseudo two-fold symmetric structure, and through homology modeling, it was predicted that BAS3 exhibited a similar conformation to ChEC88. PSI-BLAST analysis results revealed that the ChEC88 homologs were conserved across genus-spanning hemibiotrophic phytopathogens, and structural prediction supported the presence of a shared ChEC88-like motif. Functional assays confirmed that the identified homologs suppressed NLP1-induced cell death, and through site-directed mutagenesis, the ChEC88 residues critical for immune suppression were identified. Collectively, our findings reveal the conserved structural and functional properties of hemibiotrophic fungi and highlight the evolutionary significance of ChEC88-like effectors in these species.

## 2. Results

### 2.1. ChEC88 Suppresses NLP1-Induced Cell Death

ChEC88 shares 49% amino acid sequence identity with BAS3 of *P*. *oryzae* [9,17,18]. BAS3 suppresses cell death induced by transient expression of *P. *oryzae** Nep1 in *Nicotiana benthamiana* leaves [18]. We thus aimed to determine whether ChEC88 also suppresses NLP1-induced cell death in this study.

**Figure 1:**
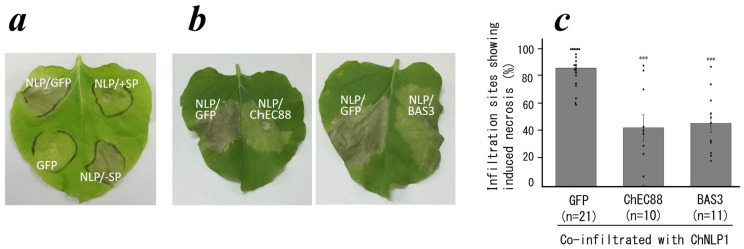
Activity of ChEC88. (**a**) Transient co-expression of ChEC88 with (+SP) or without (-SP) the signal peptide combined with cell-death-inducing elicitor ChNLP1 in *N*. *benthamiana* leaf. (**b**) Co-expression of ChEC88 or BAS3 combined with ChNLP1 in *N. benthamiana*. (**c**) Quantification of the cell-death-suppressing activities of ChEC88 and BAS3. Histograms show the sites with the percentage of necrosis. The individual dots represent the means of respective biological replicate experiments. *** indicates significant difference compared to the control site co-expressing with GFP and ChNLP1 (*** *p <* 0.001).

As shown in Figure 1 and Figure S1, ChEC88 exhibited comparable suppressive activity to BAS3. In transient expression experiments, ChEC88 suppressed ChNLP1-induced cell death when harboring a signal peptide; however, this activity was lost upon signal peptide truncation, suggesting its action in the plant apoplast. Based on these results, we further investigated the sequence–structure–function relationship of ChEC88, including NMR analysis.

### 2.2. Three-Dimensional Structure of ChEC88

We performed solution NMR analysis to determine the three-dimensional structure and assess the internal dynamics of ChEC88. As illustrated in Figure S2, mass spectrometry (MS) analysis results revealed that all ten cysteine residues in the NMR sample were present in oxidized forms, suggesting that ChEC88 contains five disulfide (SS) bonds. The ^1^H–^15^N HSQC spectrum of ^15^N-labeled ChEC88 (Figure S3) exhibited well-dispersed peaks, with the number of observable signals closely correlating with the expected number of residues, excluding five prolines. ChEC88 therefore adopted a stable conformation under the tested experimental conditions. Subsequently, we acquired a series of NMR spectra using ^15^N- and (^13^C, ^15^N)-labeled ChEC88 samples. Resonance assignment, followed by data analysis, facilitated the determination of ChEC88’s three-dimensional structure. During initial structural calculation, all ten cysteine residues were treated in their reduced forms. The preliminary three-dimensional structure indicated the formation of five SS bonds, which were subsequently incorporated as structural constraints into the final calculation. The final 20 structures were derived from 1389 NMR-based constraints. The structural statistics are summarized in Table S1.

**Figure 2:**
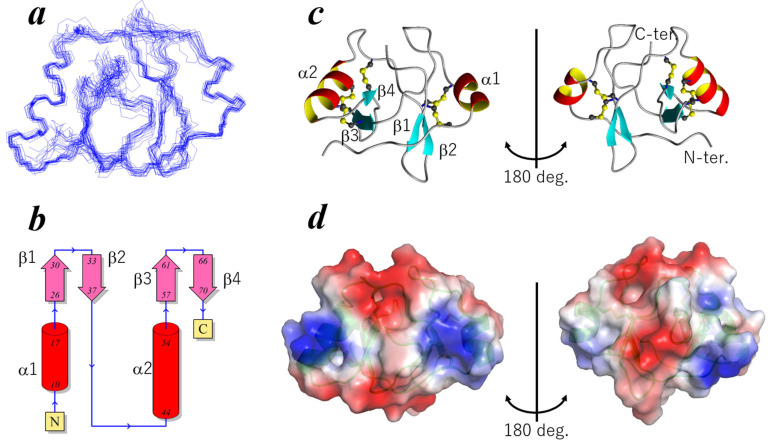
Three-dimensional structure of ChEC88 determined using NMR. (**a**) Backbone overlay of the final 20 structures. (**b**) Topology of ChEC88. The cylinder and arrow indicate the helix and strand, respectively. The number indicates the residue number. N and C indicate the N- and C-terminal ends, respectively. (**c**) Ribbon model of the representative structure. SS bonds are depicted using a ball-and-stick model. (**d**) Molecular surface electrostatic potential distribution. Red, blue, and white indicate negative, positive, and hydrophobic regions, respectively. The ribbon model is also shown in the figure.

The results presented in Figure 2a demonstrate the final ensemble of the 20 structures of ChEC88, with a root-mean-square deviation of 0.76 ± 0.18 Å for backbone atoms of residues within the secondary structural elements. As depicted in Figure 2b,c, ChEC88 consisted of two domains: an N-terminal domain (residues 1–38) and a C-terminal domain (residues 39–78). Each domain comprised a two-stranded β-sheet and an α-helix.

The N-terminal domain contained a helix, α1 (Pro10–Cys17), and an antiparallel β-sheet formed by β1 (Arg26–Ile29) and β2 (Asn34–Cys36). Similarly, the C-terminal domain included an α2 helix (Ser44–Gly54) and β3 (Asn57–Thr61) and β4 (Pro66–Ser70) strands. The two domains were stabilized by SS bonds: two in the N-terminal domain (Cys9–Cys27 and Cys17–Cys36) and three in the C-terminal domain (Cys40–Cys60, Cys46–Cys67, and Cys50–Cys69). The ^13^Cβ chemical shift values provided evidence that all cysteine residues were in their oxidized forms [19].

Notably, no disulfide bonds directly connecting the two domains were detected, suggesting that both domains were structurally autonomous. Hydrophobic residues, including Leu30, Leu35, Phe58, and Ile68, were located at the interdomain interface, possibly contributing to overall fold stabilization. Despite their low sequence identity, both domains exhibited pseudo two-fold symmetry, with a root-mean-square deviation of 4.0 Å between their Cα atoms. DALI searches revealed no structural homologs in the Protein Data Bank, suggesting that ChEC88 adopts a novel protein fold.

Electrostatic potential distribution on the ChEC88 surface is shown in Figure 2d. On one side (Figure 2d, left), the two helices exhibited relatively positively charged regions; in comparison, the top and bottom surfaces exhibited a negative electrostatic potential. The charge distribution was symmetric on this side. In contrast, the opposite side (Figure 2d, right) contained a central negatively charged patch surrounded by hydrophobic residues.

### 2.3. Dynamics and Thermal Stability of ChEC88

We investigated the dynamics of ChEC88 through NMR relaxation experiments (Figure S4). Most residues exhibited high heteronuclear NOE values (>0.6), indicating a rigid backbone. Notably, Asp39, located at the midpoint of the longest loop bridging the two domains, and C-terminal residues showed reduced heteronuclear NOE values, suggesting increased internal motion on a picosecond–nanosecond timescale. *R*_1_ relaxation rates were uniform throughout the sequence (approximately 1.3–1.4 Hz), supporting the presence of a globally stable fold. In contrast, elevated *R*_2_ values were observed at specific residues, such as Cys50, Gln53, and His55 within the C-terminal region of the α2 helix, indicating local conformational exchange on a microsecond–millisecond timescale. Anomalous *R*_2_ values of Thr15 and Val37 were possibly due to the influence of nearby dynamic side chains, namely, Thr16 and Phe59, respectively. The relatively high *R*_2_ value of Phe76 possibly also reflected side chain motion.

To evaluate its thermal stability, we recorded the one-dimensional ^1^H-NMR spectra of ChEC88 across a temperature range of 27 to 80 °C (Figure S5). Notably, ChEC88 retained its native conformation below 50 °C but underwent gradual unfolding with increasing temperature. At 80 °C, the ChEC88 structure was completely disordered. However, the original NMR spectrum reappeared upon cooling to 27 °C, indicating reversible refolding. No precipitation was observed in the NMR sample tube during thermal cycling.

### 2.4. Conservation of the ChEC88-like Motif Across Hemibiotrophic Phytopathogens

Based on the three-dimensional structure of ChEC88, we constructed a homology model of BAS3. Interestingly, the two effectors shared a similar protein fold (Figure S6), despite exhibiting less than 50% sequence identity. Moreover, both effectors exhibited a comparable ability to suppress NLP1-induced cell death (Figure 1).

**Figure 3:**
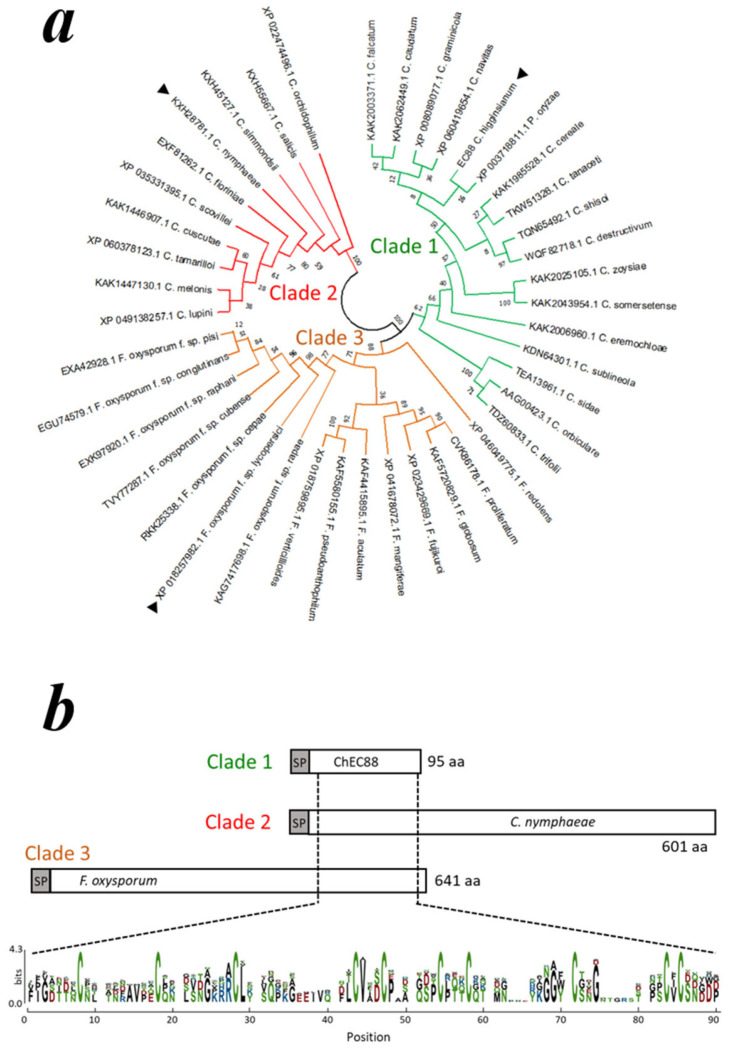
Homologous proteins of several fungal genera contain a novel conserved cysteine repeat motif. (**a**) Maximum likelihood phylogeny of the 42 amino acid sequences of ChEC88 homologous proteins found through PSI-BLAST analysis. Arrowheads indicate the representative homologs shown in (**b**). (**b**) Location of the conserved cysteine-rich repeat motif within the representative protein sequences of clades 1, 2, and 3, accompanied by a sequence logo showing the amino acid conservation pattern within this motif. Sequence logo with column heights proportional to information content (bits).

We explored additional ChEC88-like proteins with relatively low sequence similarities. PSI-BLAST search revealed that ChEC88-like proteins were conserved in *Colletotrichum*, *P. oryzae*, and *Fusarium* species. Through phylogenetic analysis, the homologs were grouped into three major clades (Figure 3a). Clade 1 comprised relatively small proteins (~100 amino acids), including ChEC88; clade 2 contained *Colletotrichum*-specific proteins; and clade 3 consisted of *Fusarium*-derived homologs. Multiple sequence alignments identified a putative conserved cysteine-rich motif containing 10 Cys residues (Figure 3b and Figure S7). Notably, the ChEC88-like domain was located at the N-terminus in clade 2 proteins and the C-terminus in clade 3 proteins.

### 2.5. Functional Conservation of ChEC88 Homologs

**Figure 4:**
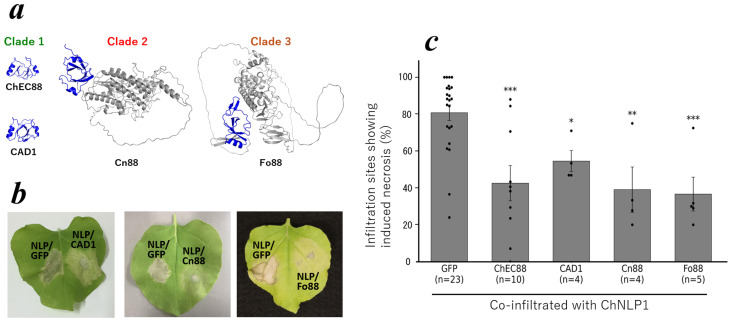
Structurally conserved region in ChEC88 and its homologous proteins across different genera share common cell-death-suppressing activity. (**a**) Structure of ChEC88 of *C. higginsianum* determined by NMR and modeled structures of its homologous proteins; CAD1 of *C. orbicular* (clade 1), Cn88 of *C. nymphaeae* (clade 2), and Fo88 of *F. oxysporum* (clade 3). The blue color indicates the conserved region used in the assay. (**b**) Representative leaves of *N. benthamiana* showing the infiltration site co-expressing ChEC88 homologous proteins from different genera with the cell-death-inducing elicitor, ChNLP1. (**c**) Quantification of the cell-death-suppressing activities of ChEC88 and its homologous proteins from different genera. The histograms show the sites with the percentage of necrosis. The individual dots represent the means of respective biological replicate experiments. * indicates significant differences compared to the control site co-expressing GFP and ChNLP1 (*** *p <* 0.001, ** *p* < 0.01, and * *p* < 0.05).

To investigate the functional conservation of ChEC88 homologs, we transiently expressed the ChEC88 representative homologs or homologous domains from each phylogenetic clade in *N. benthamiana*. Specifically, we used CAD1 (*C. orbiculare*; [20]; clade 1), the N-terminal domain of CNYM01_10604 (designated Cn88; clade 2) from *C. nymphaeae*, and the C-terminal domain of FOXG_17095 (designated Fo88; clade 3; *F. oxysporum* f. sp. *lycopersici*) for analysis. Structural models generated using AlphaFold2 confirmed the presence of ChEC88-like folds across all constructs (Figure 4a). As shown in Figure 4b,c and Figure S8, each homolog effectively suppressed ChNLP-induced cell death with similar levels of activity within the experimental error range.

Of particular note, subcellular localization predictions conducted using WoLF PSORT indicated that CAD1, BAS3, and Fo88 were predominantly secreted into the extracellular space (Table S2), consistent with their hypothesized roles as apoplastic effectors involved in immune suppression. These findings suggest that the conserved ChEC88 fold contributes to immune modulation in diverse phytopathogenic fungi via a common extracellular mechanism.

### 2.6. Identification of ChEC88 Functional Residues

**Figure 5:**
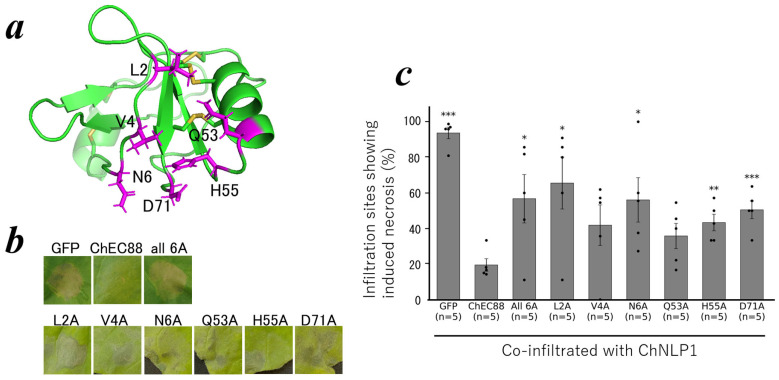
ChEC88 mutant activities. (**a**) Ribbon model structure of ChEC88. Residues introducing the Ala mutation are shown with their side chains. (**b**) Representative leaves of *N. benthamiana* showing the infiltration site co-expressing the ChEC88 mutants with the cell-death-inducer, ChNLP1. (**c**) Quantification of the cell-death-suppressing activities of the ChEC88 mutants. The histograms show the sites with the percentage of necrosis. The individual dots represent the means of respective biological replicate experiments. * indicates significant differences compared to the control site co-expressing with ChEC88 and ChNLP1 (*** *p <* 0.001, ** *p* < 0.01, and * *p* < 0.05).

To identify the residues critical for ChEC88 functions, we selected candidates based on their NMR dynamics data and three-dimensional structures. Specifically, Gln53 and His55, whose side chains were solvent-exposed, together with their neighboring residues (Leu2, Val4, Asn6, and Asp71) in the structure, were selected for Ala substitution (Figure 5a). Homology modeling showed that the three-dimensional structures of all single-point mutants and the 6A variant, in which all six residues were replaced with Ala, retained the wild-type fold (Figure S9).

We subsequently constructed plasmids for the expression of these ChEC88 variants and evaluated their activity using an agro-infiltration assay in *N. benthamiana*. Seven ChEC88 variants (L2A, V4A, N6A, Q53A, H55A, D71A, and 6A) were co-expressed with ChNLP1 to assess their ability to suppress ChNLP1-induced cell death. Infiltration assays revealed that all variants, except V4A and Q53A, exhibited significantly reduced immunosuppressive activity (Figure 5 and Figure S10). V4A and Q53A also exhibited decreased suppressive activity; however, the reduction was not statistically significant. Among the tested mutants, L2A and N6A showed a relatively greater reduction in immunosuppressive activity, comparable to that observed for the other ChEC88 variants (Figure 5 and Figure S10). These results suggest that specific residues cooperatively contribute to the immune-suppressive functions of ChEC88.

## 3. Discussion

In this study, we characterized the sequence, structure, and function of ChEC88, an effector protein of the *C. higginsianum* anthracnose fungal pathogen. We found that ChEC88 suppressed necrosis-like cell death induced by highly conserved NLPs, and the ChEC88-like proteins were broadly distributed among hemibiotrophic phytopathogens across different genera. Our findings highlight the biological significance of the ChEC88 motif in plant infections.

Solution NMR analysis results revealed that ChEC88 adopted a novel pseudo two-fold symmetric structure stabilized by five SS bonds. This conformation was reversible upon heat denaturation, indicating substantial thermal resilience. SS bonds enhance protein stability, particularly in extracellular and stress-prone environments [21]. Their frequent occurrence in effector proteins possibly reflects the evolutionary pressure to maintain structural integrity under hostile infection conditions. Previous studies [20] hypothesized that CAD1 functions as a metallothionein due to its cysteine-rich amino acid sequence. However, structural modeling indicated that all cysteine residues in ChEC88-like proteins form disulfide bonds, suggesting that these proteins are unlikely to function as metallothioneins.

Although the N- and C-terminal domains of ChEC88 exhibited low amino acid sequence identity and distinct SS bond arrangements, both adopted similar fold characteristics of the cysteine-stabilized αβ (CSαβ) motif, a structural scaffold exhibiting conformational stability and tolerance to sequence variation [22]. This architectural conservation, despite sequence divergence, suggests that the two-domain structure of ChEC88 did not originate from recent gene duplication but instead evolved via convergent stabilization into the CSαβ framework.

DALI searches using individual ChEC88 domains revealed that the N-terminal domain shared no significant structural similarity with the proteins in the Protein Data Bank, despite adopting a canonical CSαβ fold. In contrast, the C-terminal domain exhibited weak similarity (Z-score = 2.6) to tomato pistil predominant defensin 3 (TPP3), a cationic antimicrobial peptide [23]. Of particular note, TPP3 forms a CSαβ dimer superficially resembling the topology of monomeric ChEC88. However, ChEC88 was acidic, suggesting divergent functions. Although TPP3 binds phosphatidylinositol (4,5)-bisphosphate (PIP2), the target molecule of ChEC88 remains unknown.

Through bioinformatic analyses, we identified various ChEC88-like proteins in hemibiotrophic phytopathogens and classified them into three clades. Clade 1 included small proteins structurally similar to ChEC88; in comparison, clades 2 and 3 contained proteins in which the ChEC88-like domain was fused to additional C-terminal and N-terminal regions, respectively. Although all predicted structures preserved the core ChEC88 fold, the extra domains may confer additional functions, influence host specificity, or modulate the stage of infection.

ChEC88, CAD1, and BAS3, belonging to clade 1, suppress host cell death in *Nicotiana benthamiana*. Their common function and three-dimensional structure suggest that hemibiotrophic phytopathogenic fungi share a conserved immune-suppression strategy mediated by ChEC88-like proteins. Unlike effectors in clade 1, the entire function of the family members in clade 2 and 3 is unclear owing to their extra domains. A comparable example is protein disulfide isomerases (PDIs), which typically comprise catalytic **α** and **α′** domains, structural **β** and **β′** domains, a flexible **x** linker, and an acidic **C** domain involved in calcium binding. The modular organization of PDIs varies among isoforms, with some lacking specific domains, thereby reflecting their functional versatility in protein folding and cellular homeostasis [24,25].

Effectors classified into clade 1 are presumed to interact with homologous targets because of their similarity. However, effectors in clades 2 and 3 might interact with other targets due to their extra domains. Determining the specific targets of each effector in this context may clarify these mechanistic differences more precisely. Moreover, examining how these effectors function across diverse host plants could provide further insights into their mechanistic versatility and host-specific roles. Such findings may uncover the unique contributions these effectors make across various infection phases or host–pathogen interactions, revealing critical aspects of their evolutionary adaptations.

ChEC88-like proteins were widely conserved among hemibiotrophic fungi, including *Colletotrichum, Fusarium,* and *Pyricularia* species. ChEC88 expression is elevated during the biotrophic phase of infection [26]; in comparison, NLPs are predominantly expressed during the necrotrophic phase [9]. This temporal pattern suggests that ChEC88 and NLPs function sequentially to mediate the transition from biotrophy to necrotrophy during pathogenesis.

Notably, database searches for the ChEC88 cysteine-rich motif have failed to reveal homologous proteins in the obligate biotrophic powdery mildew. However, this motif is conserved across numerous hemibiotrophic *Colletotrichum* species. One exception is *C. tofieldiae*, a putative *Brassicaceae* endophyte [27], which lacked a recognizable ChEC88 homolog. Furthermore, structural homologs were restricted to a subset of hemibiotrophic species, even in structure-based searches. Despite continued efforts to define effector structural families, ChEC88 did not match any previously described effector folds [28,29]. These findings support the notion that the core effectors cannot be reliably identified based on taxonomy alone. Regardless of the primary sequence, structurally conserved motifs may constitute the fundamental determinants of effector functions and represent convergent evolutionary strategies for targeting conserved host processes.

Through NMR-guided mutagenesis, we identified the key residues contributing to ChEC88 activity. The structural reversibility revealed via NMR further confirmed the robustness of the ChEC88 fold, highlighting the importance of experimental structural biology. Our findings reiterate the significance of in vitro structural analyses, even in an era dominated by in silico modeling.

## 4. Conclusions

The results of this study demonstrate that ChEC88 is a structurally unique protein adopting a conserved fold shared by its homologs, such as BAS3. Functionally, ChEC88-like proteins suppressed host cell death and were broadly conserved among hemibiotrophic phytopathogens. The N-terminal residues of ChEC88, close to the residues with relatively large *R*_2_ values, were related to this activity. In future studies, to gain a more comprehensive understanding of ChEC88-like proteins, creating fungi with disrupted or overexpressed ChEC88 genes will be of particular interest to determine their precise functional role.

## 5. Materials and Methods

### 5.1. Activity Measurements of the ChEC88 Family and Its Mutants

To assess the biological functions of ChEC88, transient expression in *Nicotiana benthamiana* via agro-infiltration was conducted following a previously described method [9,30]. Recombinant *Agrobacterium tumefaciens* strains were cultured in LB liquid medium supplemented with appropriate antibiotics until reaching the stationary phase. Strains carrying binary vectors such as pEAQ-HT-DEST1 encoding CAD1, BAS3, Cn88, Fo88, and ChEC88 were grown in LB medium supplemented with rifampicin (50 µg/mL) and kanamycin (25 µg/mL) using *A. tumefaciens* LBA4404. For constructs harboring ChEC88-FLAG or alanine-substituted variants within pCAMBIA1301MdNcoI, *A. tumefaciens* GV3101 was cultured with kanamycin (50 µg/mL) and gentamicin (30 µg/mL). Strains carrying the p19 vector for gene-silencing suppression [31] or the GFP construct (pEAQ-HT-GFP [32]) were cultured in *A. tumefaciens* LBA4404 supplemented with kanamycin (50 µg/mL). Following three days of incubation at 28 °C, bacterial cultures were pelleted, washed, and resuspended in infiltration buffer containing 10 mM MgCl_2_, 5 mM MES (pH 5.6), and 200 µM acetosyringone. For experiments evaluating functional residues, *Agrobacterium* suspensions containing CAD1, BAS3, Cn88, Fo88, and ChEC88 constructs (OD600 = 1.0 each) were mixed with suspensions harboring the cell-death-inducing protein *Colletotrichum higginsianum* Nep1-like protein (ChNLP1: OD600 = 0.5) [9], in addition to those containing the p19 vector (OD600 = 0.1). GFP-expressing suspensions (pEAQ-HT-GFP: OD600 = 1.0), combined with ChNLP1 and p19 suspensions, served as negative controls. Mixtures of equal volumes of these suspensions were infiltrated into the abaxial side of fully expanded *N. benthamiana* leaves using a needle-less syringe.

Each experiment involved 30 injection sites and was repeated over three independent trials. To assess functional residues of ChEC88, targeted amino acid substitutions were analyzed in five independent experiments, with at least ten infiltration sites per variant per experiment. The percentage of necrotic cells at each injection site was quantified, and differences were statistically analyzed using a two-tailed Student’s *t*-test to determine significance.

### 5.2. RT-PCR Analysis to Assess Expression Level

To verify the occurrence of transient expressions at the injection sites [33], total RNA was extracted from *N*. *benthamiana* leaves at 3 days post-infiltration with *A*. *tumefaciens* strains using a Maxwell RSC Plant RNA Kit (Promega, Madison, WI, USA). First-strand cDNA was synthesized from 50 ng of total RNA using SMARTScribe Reverse Transcriptase (TaKaRa, Kusatsu, Japan) and oligo(dT) primers. RT-PCR was performed using Quick Taq HS DyeMix (TOYOBO, Osaka, Japan). The amplification program consisted of initial denaturation at 94 °C for 2 min, followed by 30 cycles of denaturation at 94 °C for 30 s, annealing at 60 °C for 30 s, and extension at 68 °C for 1 min. Negative control reactions (–RT) were performed using RNA samples without reverse transcriptase treatment. Corresponding primer sequences are listed in Table S3.

### 5.3. Preparation of Stable Isotope-Labeled Samples for NMR Analysis

An optimized DNA sequence encoding ChEC88 was designed via fusion with His x6-tag conjugated to an HRV-3C protease (TaKaRa) digestion site at the N-terminal end. Commercially synthesized DNA (GenScript, Tokyo, Japan) was introduced into the pET32b(+) vector (Merck, Darmstadt, Germany) at the VamI/EcoRi site. The vector plasmid was cloned into *E. coli* cells designated Rosetta-gami (Merck). Stable isotope-labeled NMR samples were expressed in the *E. coli* cells with M9 culture medium. For ^15^N-labeled sample preparation, we used the M9 culture medium containing ^15^NH_4_Cl (Taiyo Nippon Sanso Corporation, Tokyo, Japan) as the sole nitrogen source. For (^13^C, ^15^N)-labeled sample preparation, the culture medium contained ^13^C-glucose (Taiyo Nippon Sanso Corporation) and ^15^NH_4_Cl (Taiyo Nippon Sanso Corporation) as the sole carbon and nitrogen sources, respectively [34]. The tagged sample was crudely purified with Ni^2+^ resin (TaKaRa), and the sample solution was concentrated using an Amicon (MWCO 3000) by changing the sample buffer to 20 mM Tris-HCl (pH 8.0) containing 50 mM NaCl. After adding HRV-3C protease (Merck), the sample solution was mixed gently at 4 °C for 15 h. Tag digestion was assessed via SDS-PAGE, and the sample was purified via HPLC (SHIMADZU LC-10AD, SHIMADZU, Kyoto, Japan). Thereafter, sample purity was determined using SDS-PAGE and MS (Bruker UltrafleXtreme, Bruker, MA, USA).

### 5.4. Multidimensional NMR and Structure Determination of ChEC88

To perform NMR analysis, stable isotope-labeled ChEC88 was dissolved in H_2_O containing 50 mM NaCl and 5% D_2_O. The pH was adjusted to 6.5 with direct readings taken with a pH meter (Horiba LACUA F-2000PC, Horiba, Kyoto, Japan) using DCl and KOD. All NMR experiments were performed on a Bruker AVANCE III 800 or an AVANCE III HD 800 spectrometer. Both machines were equipped with a TCI-cryogenic probe. The sample temperature during NMR measurements was maintained at 288 K. A set of multidimensional NMR spectra (HNCA, HNCACB, CBCA(CO)NH, and ^1^H-^15^N HSQC) was recorded for backbone resonance assignment. Another set of multidimensional NMR spectra (C(CO)NH, H(CCO)NH, HCCH-TOCSY, ^1^H-^13^C CT-HSQC, and ^15^N-separarted TOCSY) was obtained for side chain resonance assignment. In addition, ^15^N-separated, ^13^C-separated, and 2D-NOESY experiments were performed to obtain distance information. The mixing time of all NOESY spectra was set at 100 ms [35].

All free induction decay (FID) data were processed with NMRPipe [36] and analyzed using Sparky/Poky [37]. Resonance assignments were completed by analyzing the heteronuclear multidimensional NMR spectra [34,35]. Thereafter, ^1^H-^1^H distance restraints were obtained from NOESY spectra. In addition, dihedral angle restraints were estimated using TALOS [38]. Hydrogen bonds in the secondary structural regions were also used as the distance restraints. These distance and angle restraints were employed for three-dimensional structure calculation. The three-dimensional structure of ChEC88 was calculated using CYANA 2.1 [39] and XPLOR-NIH 3.1 [40]. Lastly, refined structures were calculated using explicit water. Structural quality was assessed using VADAR [41] and MolProbity [42]. The final 20 structures showed no significant violations of the distance (>0.5 Å) and dihedral angle (>5°) restraints. Structural figures were generated using MOLMOL [43] and PyMol (https://github.com/schrodinger/pymol-open-source (accessed on 15 March 2024)).

### 5.5. NMR Relaxation Experiments of ChEC88

NMR relaxation experiments were performed to analyze protein dynamics using previously published pulse sequences [44]. To perform *T*_1_ relaxation analysis, 10 experiments were performed with relaxation delays of 75, 100, 150, 200, 250, 300, 400, 500, 700, and 950 ms. To perform *T*_2_ measurements, 10 experiments were performed with CPMG times of 25, 60, 80, 120, 150, 200, 300, 400, 500, and 750 ms. *T*_1_ and *T*_2_ values were estimated by fitting the peak height, *I*, using the following equation: *I* = *I*_o_ exp(−*t*/*T*_1,2_). To perform the heteronuclear NOE experiments, a 5 s recycling delay after each scan was set. NOE values were obtained by calculating the intensity ratio of the peaks recorded with and without proton saturation.

### 5.6. Bioinformatic Analysis

Homologous protein sequences of ChEC88 were identified using PSI-BLAST in the NCBI database (https://blast.ncbi.nlm.nih.gov/Blast.cgi (accessed on 23 December 2023)). After excluding redundant proteins, 42 proteins were selected. A maximum likelihood phylogenetic tree was constructed using the maximum likelihood method with Molecular Evolutionary Genetics Analysis (MEGA) software ver. 11 (https://www.megasoftware.net/ (accessed on 24 December 2023)). Amino acid sequences were aligned, and a graphical representation of the sequence conservation of amino acids (sequence logo) was generated using the CLC Main Workbench (CLC bio/Qiagen, Aarhus, Denmark).

The signal peptide cleavage site was predicted using SignalP 6.0 software (https://services.healthtech.dtu.dk/services/SignalP-6.0/ (accessed on 23 December 2023)). Three-dimensional structural prediction and homology modeling were performed using AlphaFold2 [45] and Modeller [46], respectively. Structural comparisons of proteins were performed using the DALI server (http://ekhidna2.biocenter.helsinki.fi/dali/ (accessed on 31 March 2025)).

### 5.7. Plasmid Construction for Plant Expression

To determine the potential effector functions of the representative proteins from three phylogenetic clades, full-length coding sequences (CDSs) of *CAD1* (*C. orbiculare*; NCBI Acc. No.: AAG00423.1) and *BAS3* (*P. oryzae*; XP_003718811.1) from clade 1, N-terminal 104 amino acid residues (designated Cn88) of *CNYM01_10604* (*C. nymphaeae*; KXH28781.1) from clade 2, and the region spanning residues 551 to the C-terminus of FOXG 17,095 (*F. oxysporum* f. sp. *lycopersici*; XP 018257982.1) from clade 3, which was designated as Fo88 fused with the native N-terminal 25-residue signal peptide, were amplified using the gene-specific primers listed in Table S4. Each amplified product was cloned into the pENTR/D-TOPO vector (Invitrogen, Carlsbad, CA, USA) and transferred into the pEAQ-HT-DEST1 binary expression vector [31], which drives transgene expression in plants under the control of the cauliflower mosaic virus 35S promoter, via gateway LR recombination.

To identify the amino acid residues critical for ChEC88 activity, we explored six candidate residues: L2, V4, N6, Q53, H55, and D71. A synthetic gene encoding a ChEC88 fusion protein with a C-terminal 3×FLAG tag (designated ChEC88-FLAG) and an N-terminal extracellular signal peptide was designed. Its sequence was codon-optimized for expression in *N. benthamiana* and included engineered restriction sites at both termini (IDT, Coralville, IA, USA; Table S4). In addition to wild-type ChEC88-FLAG, a variant in which all six residues were simultaneously substituted with alanine (ChEC88-FLAG 6A) was synthesized (Table S3). In addition, single-point mutants (L2A, V4A, N6A, Q53A, H55A, and D71A) were generated via PCR amplification of ChEC88-FLAG using the PrimeSTAR GXL DNA polymerase (TaKaRa) and specific primers to introduce individual alanine substitutions (Table S4). All constructs were cloned into the plant expression vector pCAMBIA1301MdNcoI [47].

The binary expression vector pEAQ-HT-DEST1 carrying *CAD1*, *BAS3*, *Cn88*, and *Fo88* was introduced into the *A. tumefaciens* LBA4404 strain via electroporation. To express wild-type ChEC88-FLAG and its alanine-substituted variants, the corresponding constructs in the pCAMBIA1301MdNcoI vector were introduced into the *A. tumefaciens* GV3101 strain using the triparental mating method [48]. Subsequently, transient expression was induced via agro-infiltration of *N. benthamiana* leaves.

## Data Availability

ChEC88 structural coordinates are available through the Protein Data Bank (https://www.rcsb.org/ (accessed on 31 March 2025)) under accession code 9UB0. The NMR chemical shifts of ChEC88 have been deposited into the Biological Magnetic Resonance Data Bank (BMRB; https://bmrb.io/ (accessed on 31 March 2025)) with data number 36751.

## Supplementary Materials

The following supporting information can be downloaded at https://www.mdpi.com/article/10.3390/plants14162562/s1. Figure S1: Gene expression analysis via RT-PCR in N. benthamiana leaves agroinfiltrated with ChEC88 and BAS3 constructs; Figure S2: MS results of 15N-labeled ChEC88; Figure S3: 1H-15N HSQC spectrum of ChEC88; Figure S4: Results of NMR relaxation experiments of ChEC88; Figure S5: 1H-NMR spectra of ChEC88 at various temperatures; Figure S6: Three-dimensional structures of BAS3 and ChEC88; Figure S7: Alignment of the amino acid sequences of ChEC88 homologous proteins found in PSI-Blast analysis; Figure S8: Gene expression analysis via RT-PCR in N. benthamiana leaves agroinfiltrated with ChEC88, CAD1, Cn88, and Fo88 constructs; Figure S9: Modeled structures of the ChEC88 mutants; Figure S10: Gene expression analysis via RT-PCR in N. benthamiana leaves agroinfiltrated with ChEC88 variant constructs; Table S1: Statistics for the final 20 NMR structures; Table S2: Predicted subcellular localization of plant-expressed CAD1, Cn88, and Fo88 proteins transiently expressed from expression vectors; Table S3: Primers used in this study; Table S4: Artificial genes used in this study.

## Abbreviations

BAK1, BRI1-associated receptor kinase 1; BAS, biotrophy-associated secreted protein; BRI1, brassinosteroid insensitive 1; CS ab, cysteine-stabilized ab; CPMG, Carr–Purcell–Meiboom–Gill; ChEC, *Colletotrichum higginsianum* effector candidate; ETI, effector-triggered immunity; ETS, effector-triggered susceptibility; FID, free induction decay; HPLC, high-performance liquid chromatography; HSQC, heteronuclear single quantum coherence; IPTG, isopropyl-β-D-thiogalactopyranoside; MS, mass spectrometry; NIS1, necrosis-inducing secreted protein 1; NLP1, Nep1-like protein; NMR, nuclear magnetic resonance; NOE, nuclear Overhauser effect; Nep1, Nep1-like protein; PTI, pattern-triggered immunity; SDS-PAGE, sodium dodecyl sulfate–polyacrylamide gel electrophoresis; TPP3, tomato pistil predominant 3 defensin.
