# Supplementary material for: Necrosis-Suppressing Effector Protein ChEC88 Adopts a Novel Structural Motif Conserved Among Genus-Spanning Hemibiotrophic Phytopathogens
Source: Plants (Basel). 2025 Aug 18;14(16):2562. doi: 10.3390/plants14162562 (PMC12389015; doi:10.3390/plants14162562)
Supplement: Supplementary file 1 [file plants-14-02562-s001.zip › Suppl_figs.pptx]

## Slide 1
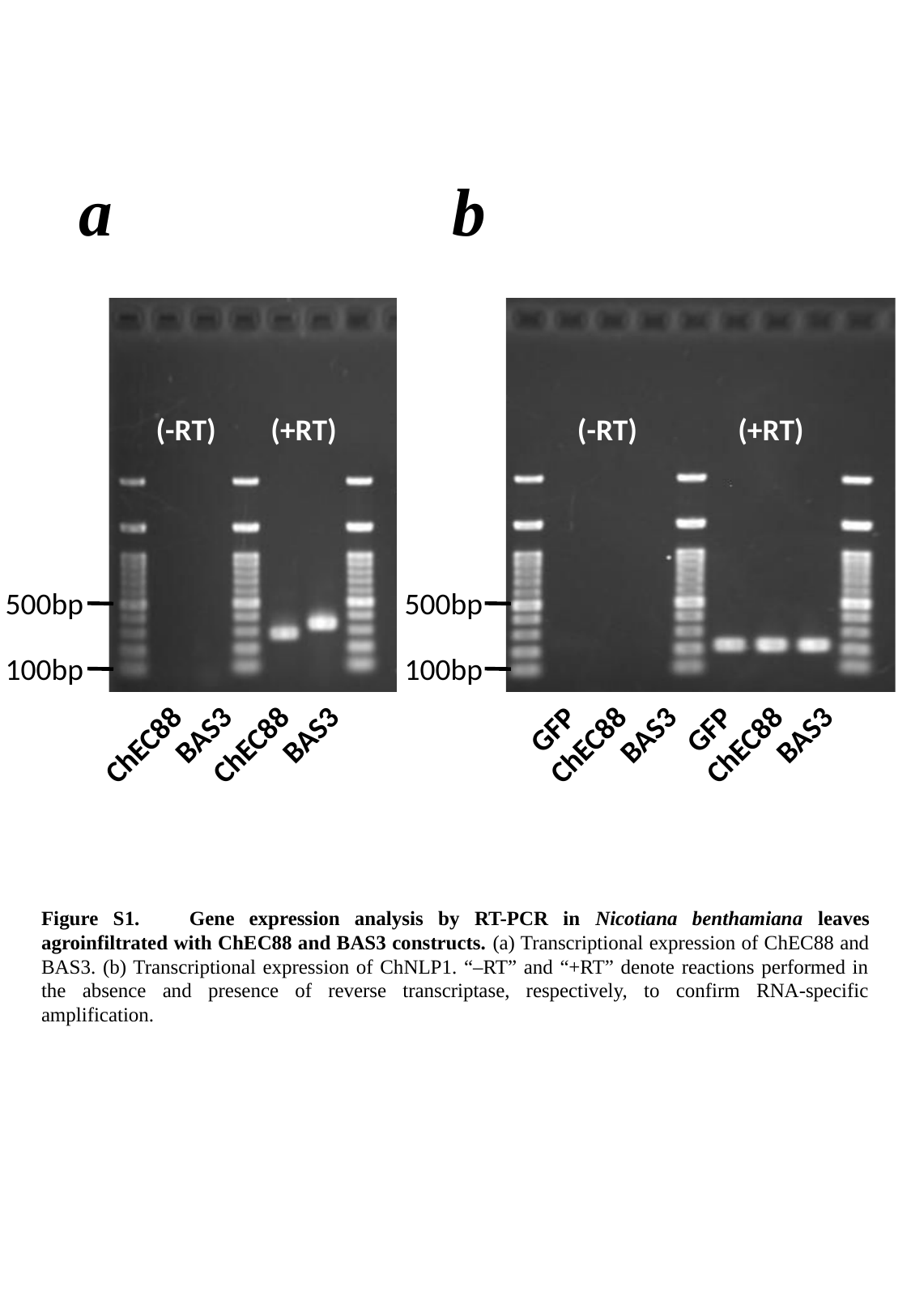

a
b
(-RT)
(+RT)
(-RT)
(+RT)
500bp
500bp
100bp
100bp
BAS3
BAS3
ChEC88
BAS3
GFP
ChEC88
BAS3
ChEC88
GFP
ChEC88
Figure S1.　Gene expression analysis by RT-PCR in Nicotiana benthamiana leaves agroinfiltrated with ChEC88 and BAS3 constructs. (a) Transcriptional expression of ChEC88 and BAS3. (b) Transcriptional expression of ChNLP1. “–RT” and “+RT” denote reactions performed in the absence and presence of reverse transcriptase, respectively, to confirm RNA-specific amplification.

## Slide 2
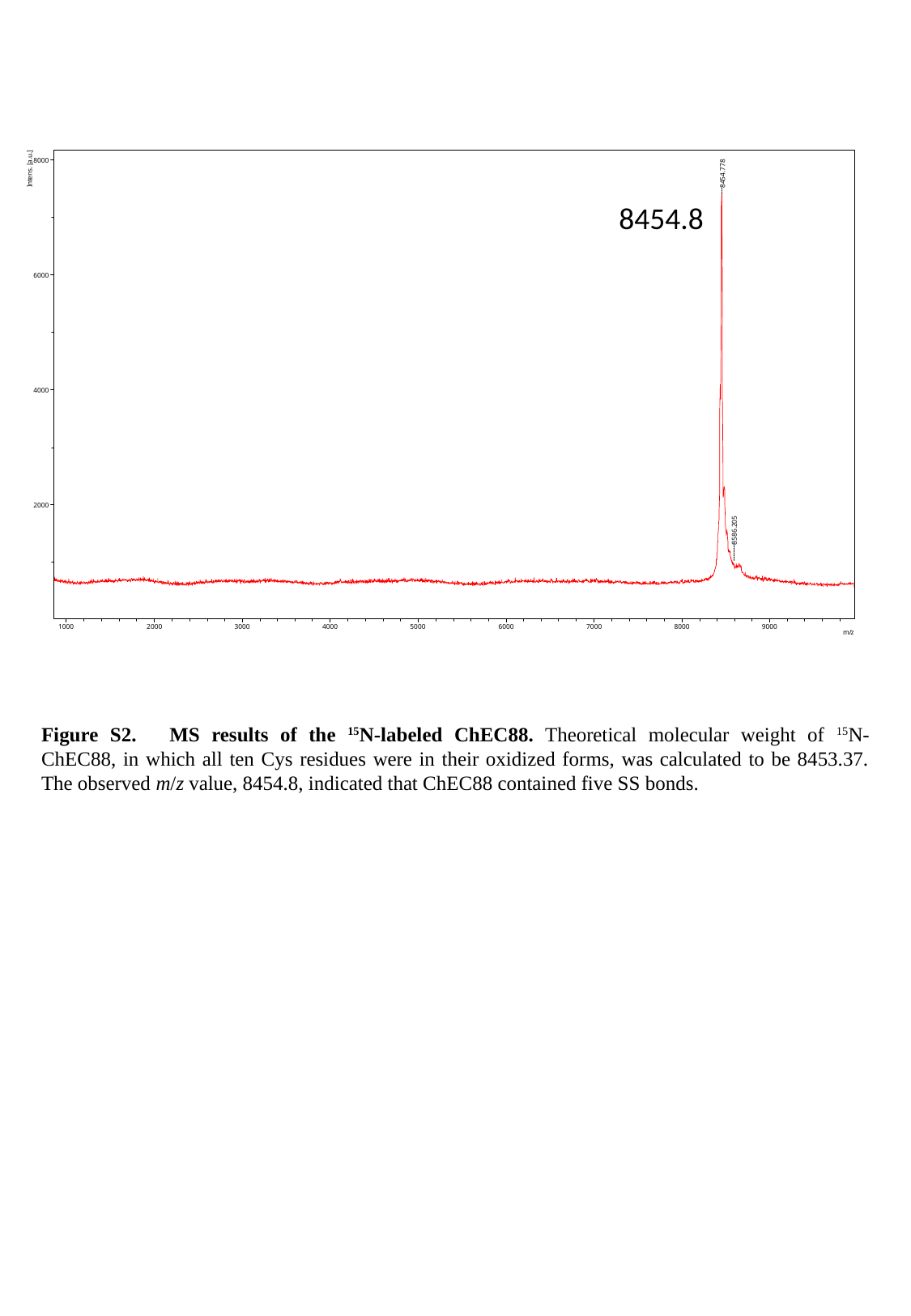

8454.8
Figure S2.	MS results of the 15N-labeled ChEC88. Theoretical molecular weight of 15N-ChEC88, in which all ten Cys residues were in their oxidized forms, was calculated to be 8453.37. The observed m/z value, 8454.8, indicated that ChEC88 contained five SS bonds.

## Slide 3
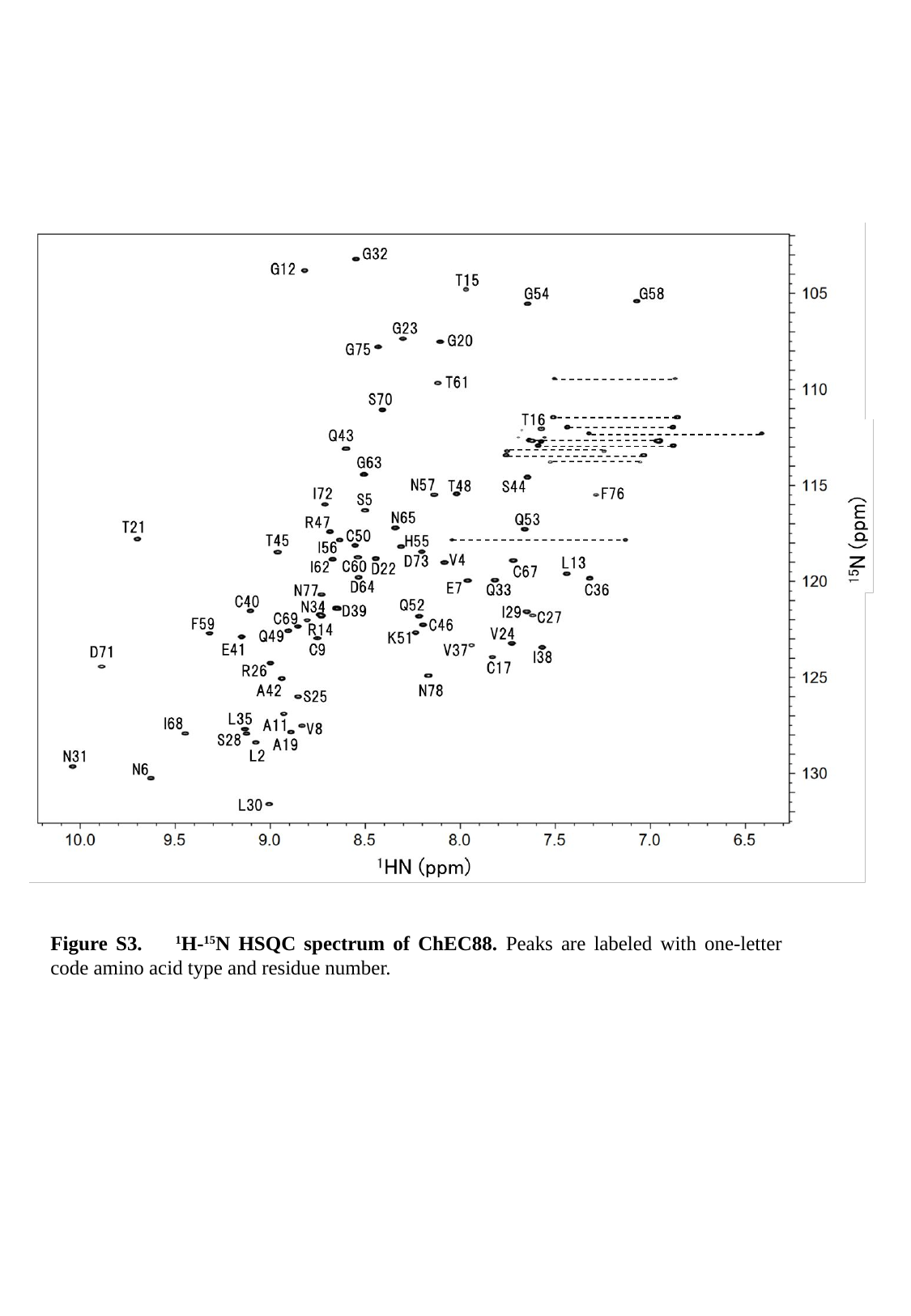

Figure S3.	1H-15N HSQC spectrum of ChEC88. Peaks are labeled with one-letter code amino acid type and residue number.

## Slide 4
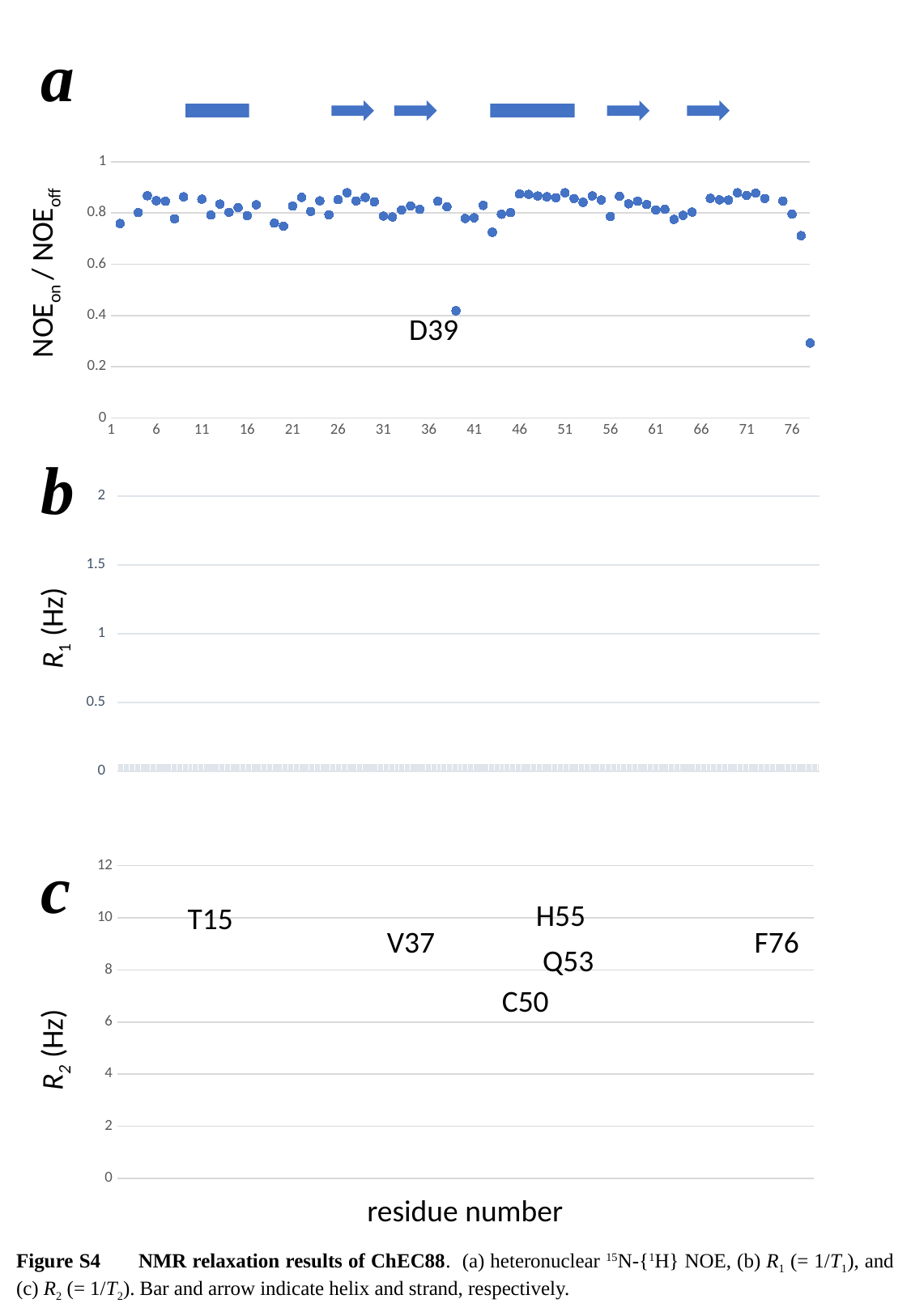

a
### Chart
| Category | |
|---|---|NOEon / NOEoff
D39
b
### Chart
| Category | |
|---|---|
| 1 | None |
| 2 | 1.34636 |
| 3 | None |
| 4 | 1.34636 |
| 5 | 1.36763 |
| 6 | 1.38759 |
| 7 | 1.37349 |
| 8 | 1.36377 |
| 9 | 1.42795 |
| 10 | None |
| 11 | 1.46318 |
| 12 | 1.45677 |
| 13 | 1.40224 |
| 14 | 1.43213 |
| 15 | 1.43113 |
| 16 | None |
| 17 | 1.48183 |
| 18 | None |
| 19 | 1.31336 |
| 20 | 1.33791 |
| 21 | 1.37493 |
| 22 | 1.47055 |
| 23 | 1.43258 |
| 24 | 1.39382 |
| 25 | 1.32803 |
| 26 | 1.37833 |
| 27 | 1.42052 |
| 28 | 1.45217 |
| 29 | 1.4442 |
| 30 | 1.40784 |
| 31 | 1.43622 |
| 32 | 1.36135 |
| 33 | 1.34246 |
| 34 | 1.34143 |
| 35 | 1.30429 |
| 36 | None |
| 37 | 1.35043 |
| 38 | 1.43148 |
| 39 | 1.22525 |
| 40 | 1.37135 |
| 41 | 1.39305 |
| 42 | 1.42536 |
| 43 | 1.33525 |
| 44 | 1.34574 |
| 45 | 1.43862 |
| 46 | 1.51444 |
| 47 | 1.4639 |
| 48 | 1.43316 |
| 49 | 1.48825 |
| 50 | 1.41632 |
| 51 | 1.51344 |
| 52 | 1.4477 |
| 53 | 1.35807 |
| 54 | 1.39675 |
| 55 | 1.36267 |
| 56 | 1.36234 |
| 57 | 1.52333 |
| 58 | 1.3702 |
| 59 | 1.3845 |
| 60 | 1.39353 |
| 61 | 1.32153 |
| 62 | 1.29485 |
| 63 | 1.44981 |
| 64 | 1.26737 |
| 65 | 1.29405 |
| 66 | None |
| 67 | 1.4904 |
| 68 | 1.40829 |
| 69 | 1.44876 |
| 70 | 1.38634 |
| 71 | 1.40979 |
| 72 | 1.47321 |
| 73 | 1.34835 |
| 74 | None |
| 75 | 1.44327 |
| 76 | 1.38934 |
| 77 | 1.36993 |
| 78 | 1.25579 |R1 (Hz)
c
### Chart
| Category | |
|---|---|
| 1 | 0.0 |
| 2 | 9.46679 |
| 3 | 0.0 |
| 4 | 8.02263 |
| 5 | 8.78773 |
| 6 | 9.89962 |
| 7 | 8.97454 |
| 8 | 11.6826 |
| 9 | 9.96864 |
| 10 | 0.0 |
| 11 | 9.41534 |
| 12 | 13.1648 |
| 13 | 8.97646 |
| 14 | 9.77077 |
| 15 | 20.242 |
| 16 | 0.0 |
| 17 | 8.70583 |
| 18 | 0.0 |
| 19 | 8.35853 |
| 20 | 7.41967 |
| 21 | 8.46359 |
| 22 | 9.91321 |
| 23 | 7.99272 |
| 24 | 8.02955 |
| 25 | 10.2462 |
| 26 | 8.13081 |
| 27 | 9.86105 |
| 28 | 9.69174 |
| 29 | 8.79058 |
| 30 | 9.04964 |
| 31 | 8.40287 |
| 32 | 8.51497 |
| 33 | 8.82485 |
| 34 | 8.06075 |
| 35 | 7.21544 |
| 36 | 0.0 |
| 37 | 17.7692 |
| 38 | 10.3726 |
| 39 | 5.55313 |
| 40 | 7.79756 |
| 41 | 8.22595 |
| 42 | 6.84654 |
| 43 | 8.12382 |
| 44 | 8.7112 |
| 45 | 8.54285 |
| 46 | 8.38203 |
| 47 | 8.82831 |
| 48 | 8.72701 |
| 49 | 8.3432 |
| 50 | 12.5686 |
| 51 | 9.55206 |
| 52 | 9.07483 |
| 53 | 14.9784 |
| 54 | 8.96846 |
| 55 | 21.5089 |
| 56 | 9.13659 |
| 57 | 10.4089 |
| 58 | 9.30812 |
| 59 | 8.0763 |
| 60 | 9.69719 |
| 61 | 9.61832 |
| 62 | 9.37081 |
| 63 | 7.22496 |
| 64 | 7.95235 |
| 65 | 9.35406 |
| 66 | 0.0 |
| 67 | 10.953 |
| 68 | 8.50936 |
| 69 | 9.46458 |
| 70 | 8.01142 |
| 71 | 7.64753 |
| 72 | 10.7104 |
| 73 | 12.9501 |
| 74 | 0.0 |
| 75 | 8.18412 |
| 76 | 16.768 |
| 77 | 6.84119 |
| 78 | 3.25762 |H55
T15
V37
F76
Q53
C50
R2 (Hz)
residue number
Figure S4	NMR relaxation results of ChEC88. (a) heteronuclear 15N-{1H} NOE, (b) R1 (= 1/T1), and (c) R2 (= 1/T2). Bar and arrow indicate helix and strand, respectively.

## Slide 5
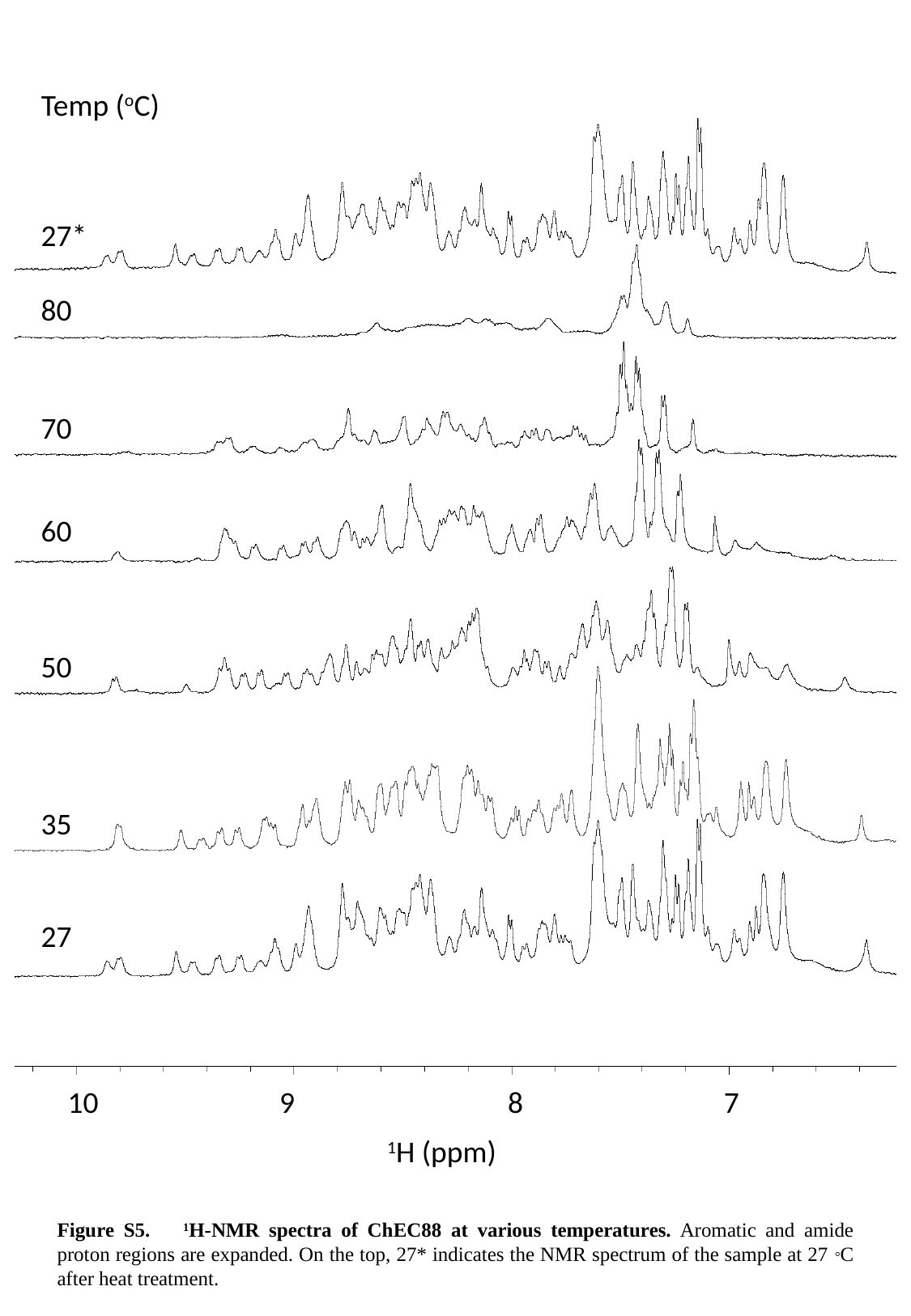

Temp (oC)
27*
80
70
60
50
35
27
10
9
8
7
1H (ppm)
Figure S5.	1H-NMR spectra of ChEC88 at various temperatures. Aromatic and amide proton regions are expanded. On the top, 27* indicates the NMR spectrum of the sample at 27 °C after heat treatment.

## Slide 6
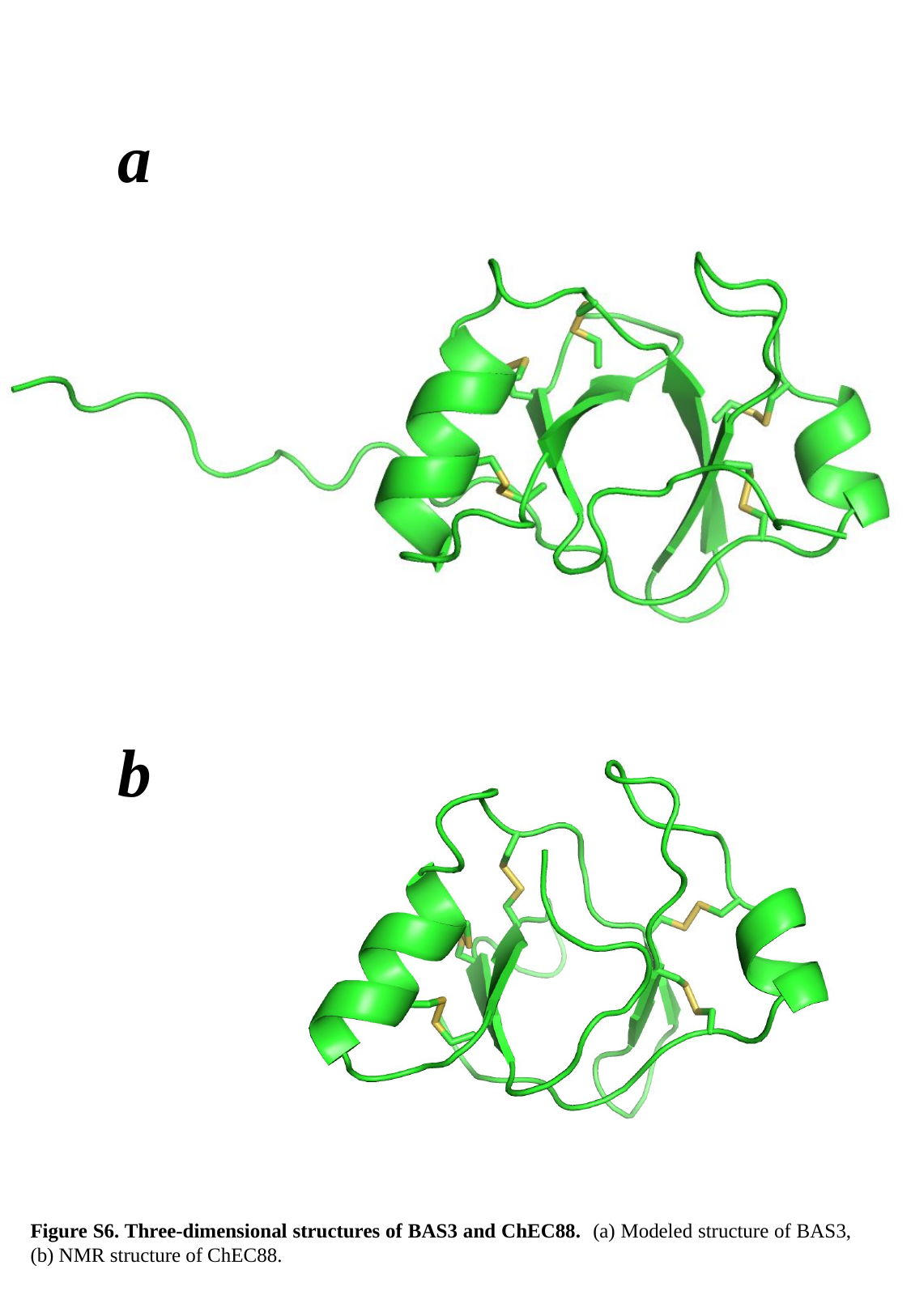

a
b
Figure S6. Three-dimensional structures of BAS3 and ChEC88. (a) Modeled structure of BAS3, (b) NMR structure of ChEC88.

## Slide 7
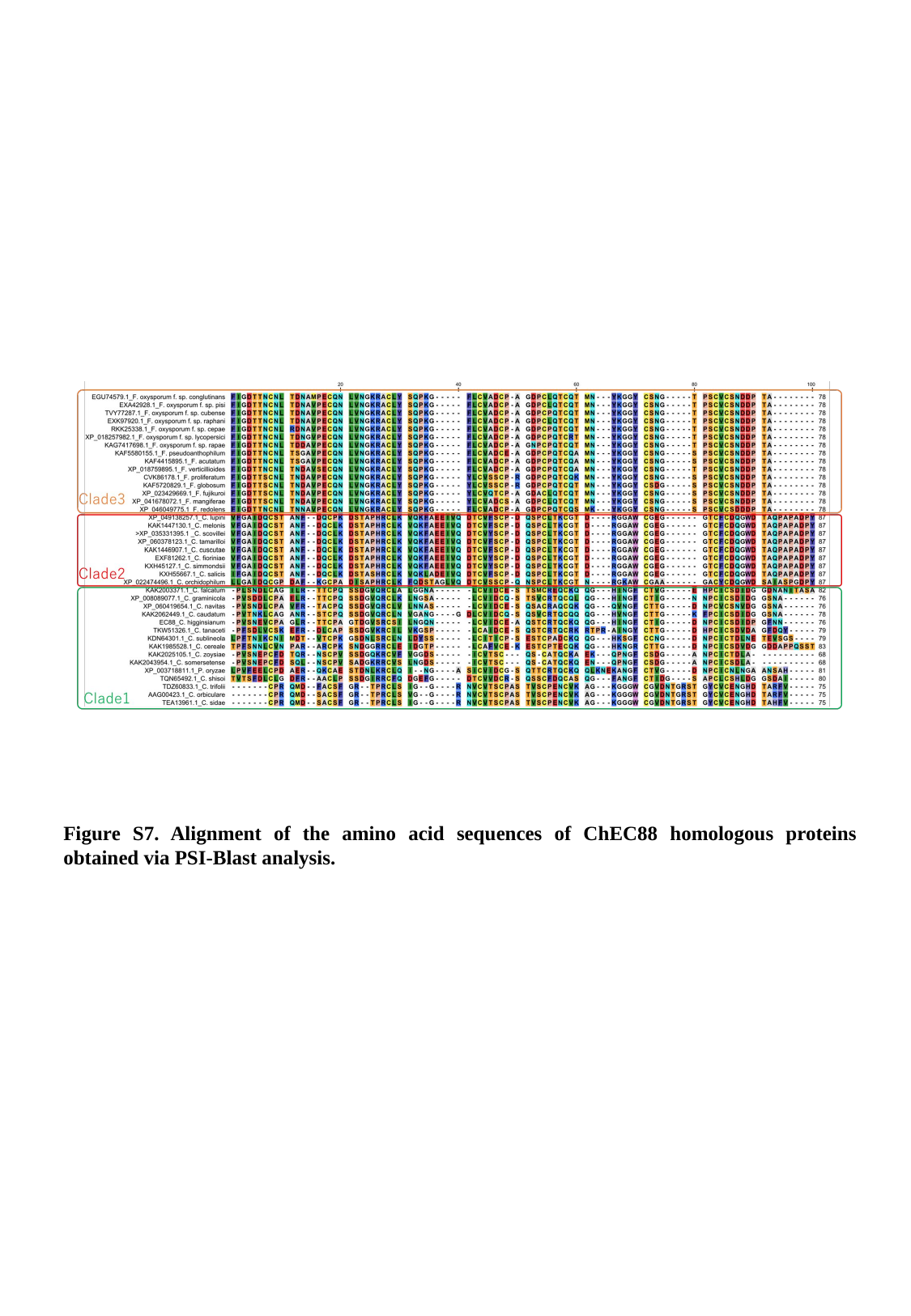

Figure S7. Alignment of the amino acid sequences of ChEC88 homologous proteins obtained via PSI-Blast analysis.

## Slide 8
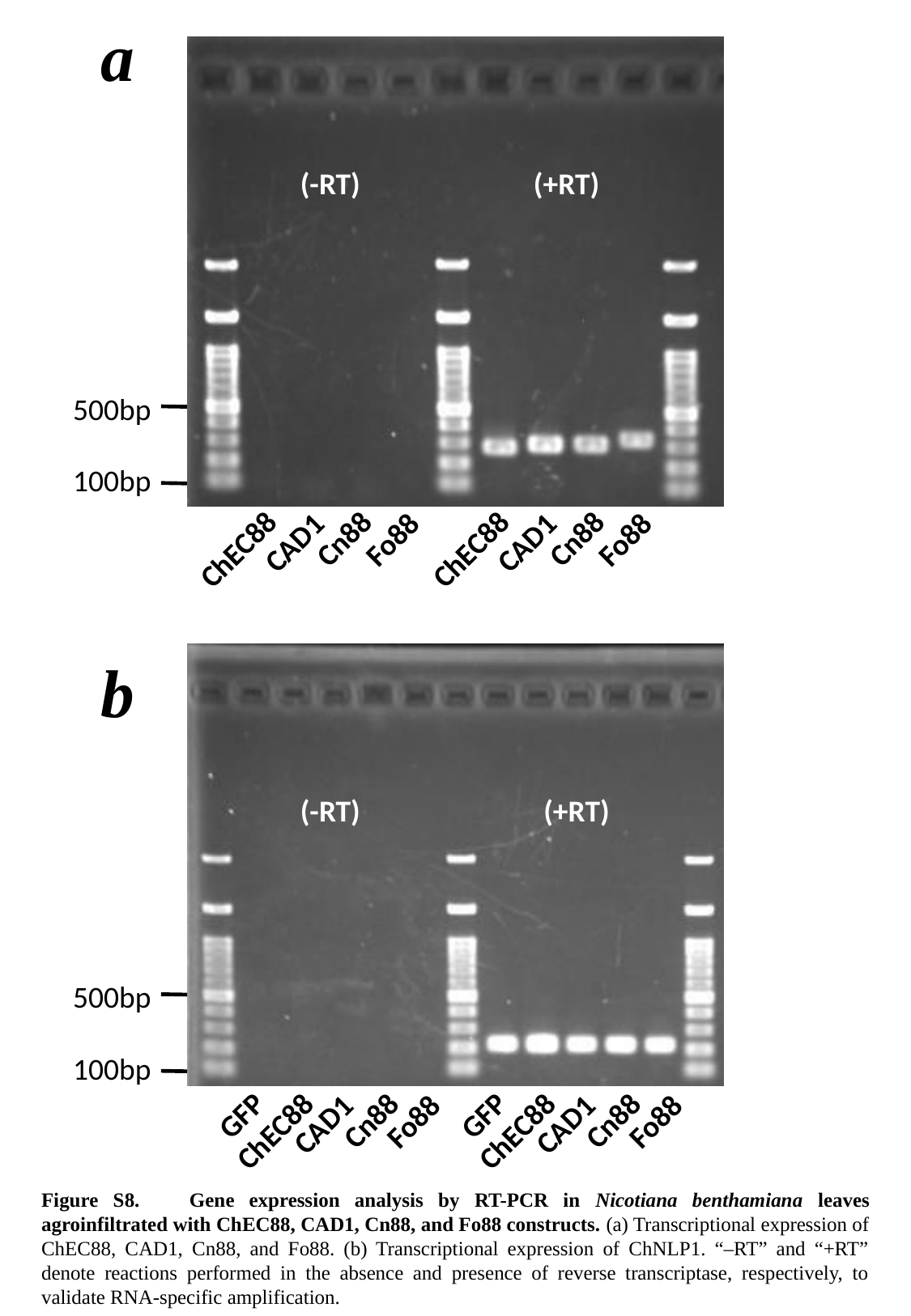

a
(-RT)
(+RT)
500bp
100bp
ChEC88
Cn88
ChEC88
Cn88
Fo88
Fo88
CAD1
CAD1
b
(-RT)
(+RT)
500bp
100bp
GFP
ChEC88
Cn88
GFP
ChEC88
Cn88
CAD1
Fo88
CAD1
Fo88
Figure S8.　Gene expression analysis by RT-PCR in Nicotiana benthamiana leaves agroinfiltrated with ChEC88, CAD1, Cn88, and Fo88 constructs. (a) Transcriptional expression of ChEC88, CAD1, Cn88, and Fo88. (b) Transcriptional expression of ChNLP1. “–RT” and “+RT” denote reactions performed in the absence and presence of reverse transcriptase, respectively, to validate RNA-specific amplification.

## Slide 9
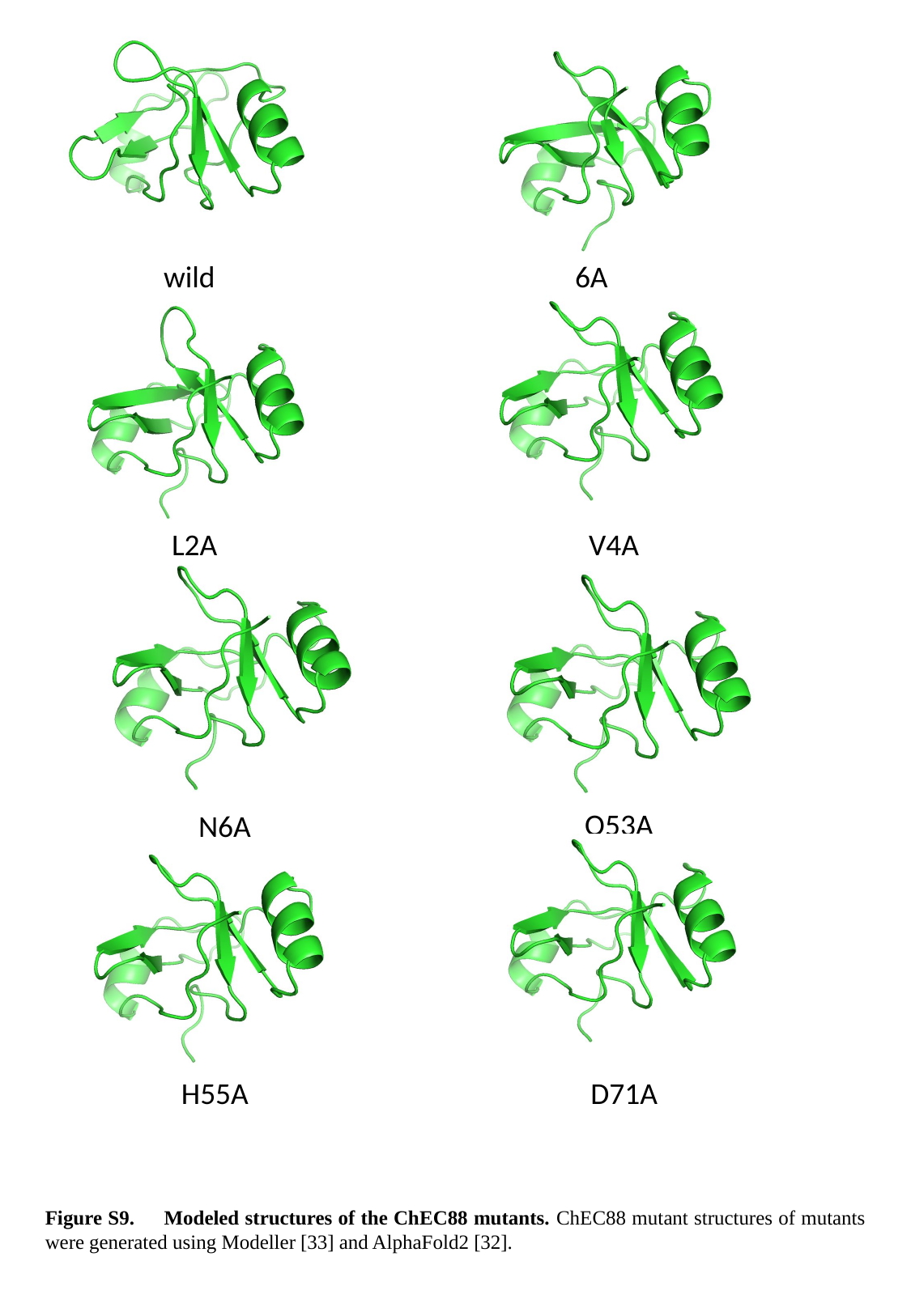

wild
6A
L2A
V4A
Q53A
N6A
H55A
D71A
Figure S9. Modeled structures of the ChEC88 mutants. ChEC88 mutant structures of mutants were generated using Modeller [33] and AlphaFold2 [32].

## Slide 10
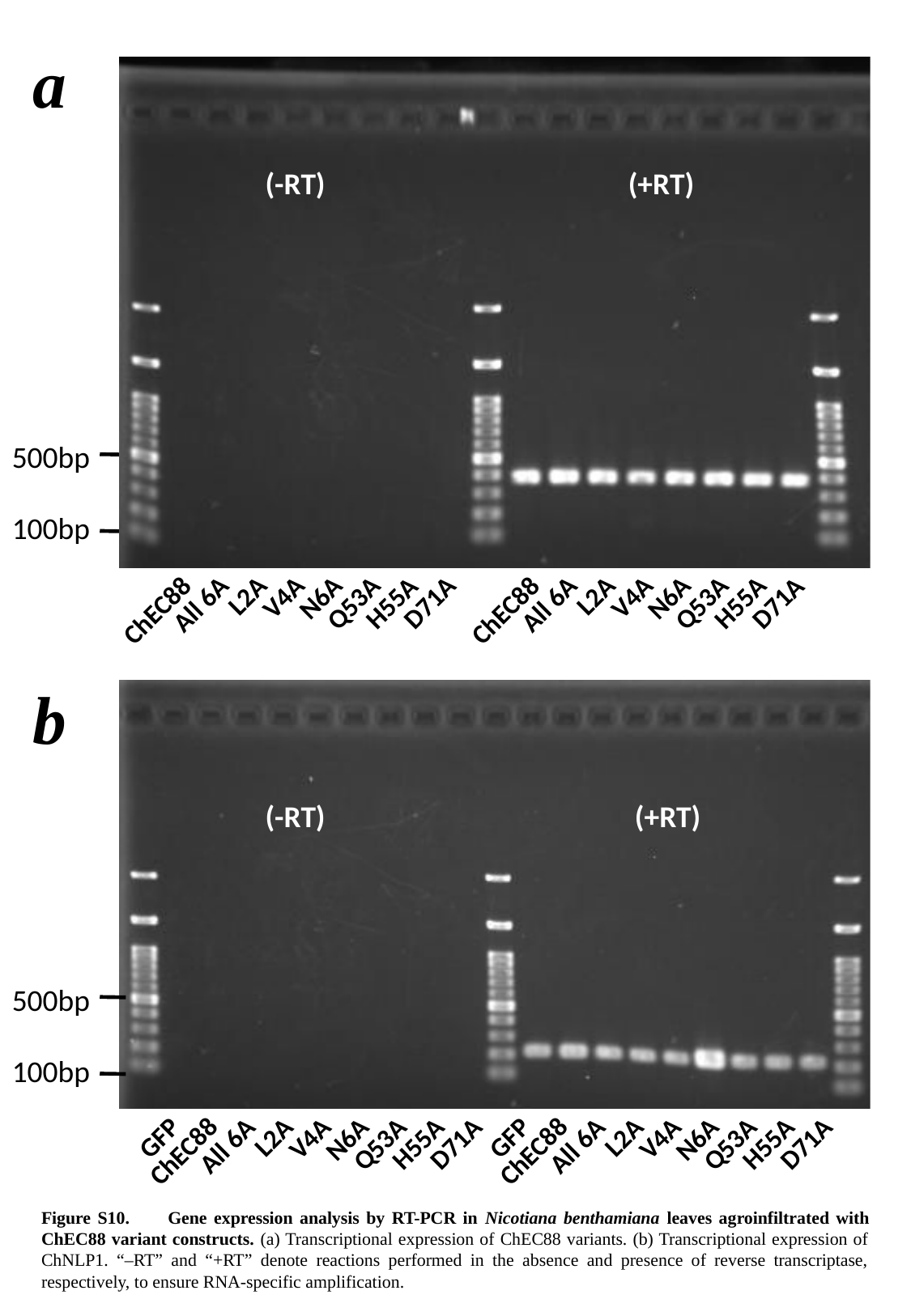

a
(-RT)
(+RT)
500bp
100bp
Q53A
H55A
D71A
Q53A
H55A
D71A
ChEC88
All 6A
L2A
V4A
N6A
ChEC88
All 6A
L2A
V4A
N6A
b
(-RT)
(+RT)
500bp
100bp
Q53A
H55A
D71A
Q53A
H55A
D71A
GFP
ChEC88
All 6A
L2A
V4A
N6A
GFP
ChEC88
All 6A
L2A
V4A
N6A
Figure S10.　 Gene expression analysis by RT-PCR in Nicotiana benthamiana leaves agroinfiltrated with ChEC88 variant constructs. (a) Transcriptional expression of ChEC88 variants. (b) Transcriptional expression of ChNLP1. “–RT” and “+RT” denote reactions performed in the absence and presence of reverse transcriptase, respectively, to ensure RNA-specific amplification.
